# New Communities on Eucalypts Grown Outside Australia

**DOI:** 10.3389/fpls.2016.01812

**Published:** 2016-11-29

**Authors:** Sarah Mansfield

**Affiliations:** Faculty of Agriculture and Environment, University of Sydney, CamperdownNSW, Australia

**Keywords:** biological control, *Eucalyptus*, herbivore guilds, invasive species, parasitoids, predators

## Abstract

The expansion of eucalypt forestry worldwide has been accompanied by accidental and deliberate introductions of Australian insects associated with eucalypts. Local insect species have also colonized introduced eucalypts in many regions. This situation provides a unique opportunity to observe the development of new insect communities across trophic levels. Here the history of Australian invaders and native colonizers on eucalypts outside Australia is reviewed from the perspective of herbivore guilds: leaf chewers, sap suckers, wood borers, gall formers, termites. Historical patterns of invasion are identified across these guilds. Very few species of Australian leaf chewers, wood borers or termites have become widespread but these guilds are proportionately high in native colonizers. In contrast, sap suckers have multiple invasive species globally with relatively fewer native colonizers. The gall former guild also has several invasive species but so far includes no native colonizers, perhaps due to their tendency to have highly specific host plant associations. Natural enemies of Australian invaders are also members of new eucalypt insect communities, partly through planned biological control programs, but the rate of accidental introductions at higher trophic levels is increasing steadily. At the same time, local natural enemies enter eucalypt communities either to form new associations with Australian invaders or to follow native colonizers into this new habitat. Australian sap suckers have attracted far more new associations than other guilds so far. Native leaf chewers have often been followed by their local natural enemies into eucalypt communities, particularly in Brazil. Generally there are fewer records relating to local natural enemies and their role in new eucalypt communities. The complexity of new eucalypt communities outside Australia is expected to increase in future.

## Introduction

The worldwide expansion of eucalypt forestry (genera *Angophora*, *Corymbia* and *Eucalyptus*, Hui et al., 2014) has been accompanied by accidental introductions of Australian insect herbivores associated with eucalypts. Outside of their endemic geographic range (Australia for the majority of species, Wardell-Johnson et al., 1997), eucalypts represent distinct ecological communities that are vulnerable to more exotic arrivals. This dynamic situation provides a unique opportunity to observe the establishment of new insect communities across trophic levels in eucalypt plantations on a global scale. Recent work has used eucalypt communities to explore the characteristics of invasive species (Nahrung and Swain, 2015) and to evaluate the risk to Australian eucalypt plantations from invasion by exotic insect pests (Paine et al., 2011).

Beyond Australia, new invertebrate communities found in eucalypt forests elsewhere are formed not only through the invasion of Australian eucalypt specialist species but also through the colonization by local insect species (Ohmart and Edwards, 1991; Nair, 2007; Paine et al., 2011). The number of native species colonizing an exotic plant species increases with time after introduction (Brandle et al., 2008) so the potential for interspecific competition between Australian invaders and native colonizers on eucalypts should also increase with time. Competition would, in theory, be more intense between species from within the same herbivore guild than between those from different guilds (Denno et al., 1995). However, tests of this hypothesis have led to mixed results (Kaplan and Denno, 2007). The guild composition of Australian invaders has been described in exotic eucalypt stands in some regions (Withers, 2001; Paine et al., 2010) but the guild composition of native colonizers has received less attention.

The arrival of Australian invertebrate invaders often has been followed by the deliberate introduction of their co-evolved natural enemies to provide biological control (Garnas et al., 2012). Generalist predators and parasitoids that are native to the region may also form new associations with the Australian invaders (Santana and Burckhardt, 2007; Protasov et al., 2008). Similarly, native colonizers of eucalypts may be followed by their native natural enemies into the new community (de Oliveira et al., 2000; De Menezes et al., 2013). These processes ensure a gradual accumulation of species at higher trophic levels. The question is: have all of the herbivore guilds gained natural enemies to a similar degree?

In this contribution the history of eucalypt insect communities that have developed outside Australia is reviewed from the perspective of herbivore guilds, including both the Australian invaders and native colonizers, to compare the relative success of these two groups across different guilds. The accumulation of natural enemies (including deliberate introductions for biological control, accidental arrivals, and new associations) is also compared between the herbivore guilds in these new communities to determine if some guilds have acquired more natural enemies than others.

## Records of Eucalypt Insects

The published literature (up to June 2015) was searched using the Web of Science (all years and databases included: Web of Science, Current Contents, BIOSIS Previews, CAB Abstracts, Agricola). Also searched were the CABI Forestry and Invasive species compendia for all records of insects associated with eucalypts. From these records, a database of insect herbivores associated with eucalypts outside of Australia was prepared (**Supplementary Material**). This database included records of the country/countries showing an insect’s association with eucalypts, the insect’s country or region of origin, and in the case of Australian invaders, the year of the first record in each country was noted. The year of publication was used if the actual year of record could not be determined from the text. No attempt was made to differentiate between invasions that originated from Australia versus secondary dispersal by Australian invaders from their new areas of establishment into adjacent countries. For natural enemies associated with eucalypt herbivores, the prey or host species was also recorded. Generalist insect herbivores that are not confined to eucalypts in their native Australian range but instead feed on a broad range of plants that happens to include eucalypts were not included in further analysis.

## Guild Definitions

Eucalypt herbivores, irrespective of geographic origin, were grouped into the following feeding guilds: leaf chewers, sap suckers, gall formers, wood borers, termites, and others. Leaf chewers were chewing herbivores, mostly Australian and native Coleoptera and Lepidoptera, and native leaf cutting ants that have colonized introduced eucalypts in Central and South America (Speight and Wylie, 2001). Also included in this guild were two Australian eucalypt leaf miners (*Phylacteophaga froggatti* and *Acrocercops laciniella*) that have invaded New Zealand (Withers, 2001). Sap suckers (Hemiptera) included Australian psyllids as well as native species that colonized introduced eucalypts. Wood borers (mostly Coleoptera) were restricted to those that infest living trees; not included were wood boring species that feed only on dead logs or harvested timber. Various species of termites may feed on living trees (Pearson et al., 2010), tree roots (Zhenghong, 2003; Nair, 2007) or dead wood (Bain and Jenkin, 1983). Classification of termites in this study was problematic because it was not always possible to determine the appropriate guild for each termite species based on the available literature. Thus termites were treated as a separate group. Root feeders (other than termites) were not included as a separate guild because no Australian root feeders specific to eucalypts have been recorded as invaders. Native colonizers categorized as ‘other’ included generalist root feeders [often scarab larvae that attack seedlings (Zondag, 1979; Zhenghong, 2003)] and herbivores that could not be assigned accurately to a specific guild from the information available. Two Australian species were also categorized as ‘other’: a nectar feeding fly (Tribe et al., 1989) and a seed feeding thrips (Mound and Walker, 2012). These ‘other’ herbivores were excluded from further analysis.

## Australian Invaders

**FIGURE 1:**
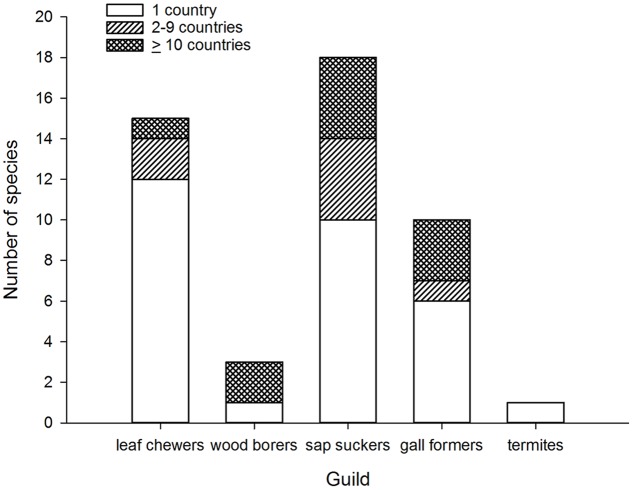
**Number of invasive species by number of invaded countries grouped according to guild of Australian eucalypt insects**.

**FIGURE 2:**
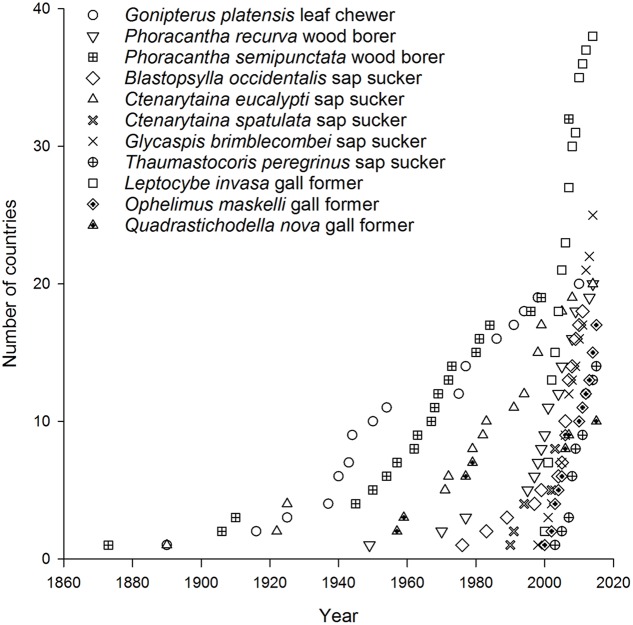
**Timeline for invasion by the 11 most widespread Australian eucalypt insects**.

Today there are more than 40 Australian insect species associated with eucalypts that occur in at least one country beyond Australia (Withers, 2001; Paine et al., 2010) with 11 species now found in multiple countries. A further 29 species have reached just one country so far (**Figure 1**) (CABI, 2015a,b). Of the 11 most invasive eucalypt herbivores, the earliest began to disperse in the late 1800s. These included a leaf feeder *Gonipterus platensis* (previously known as *G. scutellatus* but now recognized as a cryptic species complex, Mapondera et al., 2012), a wood borer *Phoracantha semipunctata*, and a sap sucker *Ctenarytaina eucalypti* (Withers, 2001) (**Figure 2**). These species were followed in the mid-1900s by a second wood borer *Phoracantha recurva* (Drinkwater, 1975) and the first gall former *Quadrastichodella nova* (Timberlake, 1957). Thereafter a cluster of four sap suckers and two gall formers dispersed rapidly worldwide, beginning with *Blastopsylla occidentalis* and ending with *Thaumastocoris peregrinus*, which is the most recent Australian invader (Withers, 2001; Mendel et al., 2004, 2007; Noack and Coviella, 2006; Paine et al., 2010). Paine et al. (2011) identified two historical phases of movement for Australian eucalypt insects: early establishment was 1873–1955 and late establishment was after 1955. Certainly more recent invasions have spread faster than earlier events (this is evidenced in the timelines for *P. semipunctata* versus *Leptocybe invasa*, **Figure 2**). This trend is apparent even within the same guild. For example, it took more than 90 years for the first sap sucker, *C. eucalypti*, to disperse to ten countries whereas the most recent sap sucker invasion by *T. peregrinus* took only 8 years (**Figure 2**). Faster transportation rates, intensification of trade and the global expansion of eucalypt plantations have all contributed to the increasing speed of invasion events (Wingfield et al., 2008; Evans, 2009). These changes have increased the probability of invaders surviving the journey and finding suitable habitat upon arrival.

When broken down by guild, two patterns of colonization by Australian eucalypt insects become apparent. The first pattern shows a small number of dominant species within the guilds that are widespread globally; other guild members are only found in a restricted range outside of Australia. For example, two congeneric wood borers, the cerambycids *P. semipunctata* and *P. recurva*, have spread widely outside Australia (Paine and Millar, 2002) whereas the total number of invasive eucalypt wood borer species is low compared with other guilds (**Figure 1**). The implementation of biosecurity regulations has presumably restricted movement of wood borers, including termites, out of Australia (Commonwealth of Australia, 2015; Sopow et al., 2015) despite continued use of wooden packaging materials for shipping in many parts of the world (Rassati et al., 2015). The Australian termite, *Porotermes adamsoni*, attacks live eucalypts but is subject to eradication where it has established colonies in New Zealand (Pearson et al., 2010). Several generalist Australian termite species are now found in New Zealand (Bain and Jenkin, 1983; Withers, 2001; Philip et al., 2008) but were not included in this analysis. Among leaf chewers, only the *G. platensis* species complex is widespread with most other members of this guild being found in just one country outside Australia (often New Zealand). The lack of widespread invasion by most leaf chewers is not a reflection of narrow host ranges among guild members. For example, the eucalypt chrysomelid, *Paropsis charybdis*, colonized New Zealand in the early 1900s and has been recorded from >60 eucalypt species (CABI, 2015a) yet has not spread to any other eucalypt growing countries so far. More recent arrivals to New Zealand from the leaf chewer guild include *Uraba lugens* and *Phylacteophaga froggatti*, which both attack a wide range of eucalypts (Potter and Stephens, 2005; CABI, 2015a). Two leaf chewing species with potential for further spread are the eucalypt chrysomelids *Trachymela sloanei*, which has spread to New Zealand, South Africa and the USA (Withers, 2001; Paine et al., 2010), and *Paropsisterna selmani*, the first paropsine to reach Europe (Reid and De Little, 2013).

The second, more dispersive pattern, in contrast, has multiple invasive species within the same guild. This is demonstrated by the five species of sap suckers (including four psyllids) that have colonized 8–20 countries outside of Australia, with most of this expansion having taken place over the past 30 years (Santana and Burckhardt, 2007; Sopow et al., 2012). The sap sucker *B. occidentalis* was the second Australian species associated with eucalypts to invade China (Yen et al., 2013). Those sap suckers with more restricted distributions are often found in New Zealand (Bejakovich, 2002; Martoni et al., 2016) although a new invasive species, *Platyobria biemani*, was recently found in Greece (Burckhardt et al., 2014). Gall formers also demonstrate this more dispersive pattern. The first member of this guild to become invasive was *Q. nova*, which now occurs in at least seven countries (Kim and La Salle, 2008; Paine et al., 2010). From 1999 a further nine species of gall formers have been found outside Australia for the first time (Berry and Withers, 2002; Protasov et al., 2007; Kim and La Salle, 2008) however, later work has suggested two of these species may be parasitoids, not gall formers (Klein et al., 2015). One species, *L. invasa*, has spread to 39 countries since 2000, the fastest invasion by any Australian insect associated with eucalypts (Garnas et al., 2012; Nugnes et al., 2015). This gall former was the first Australian eucalypt insect to enter China (Zhu et al., 2012) and India (Ramanagouda et al., 2010). Another species, *Ophelimus maskelli*, is recorded from 13 countries so far (Doganlar and Mendel, 2007; Branco et al., 2009; Burks et al., 2015b).

## Native Colonizers

**FIGURE 3:**
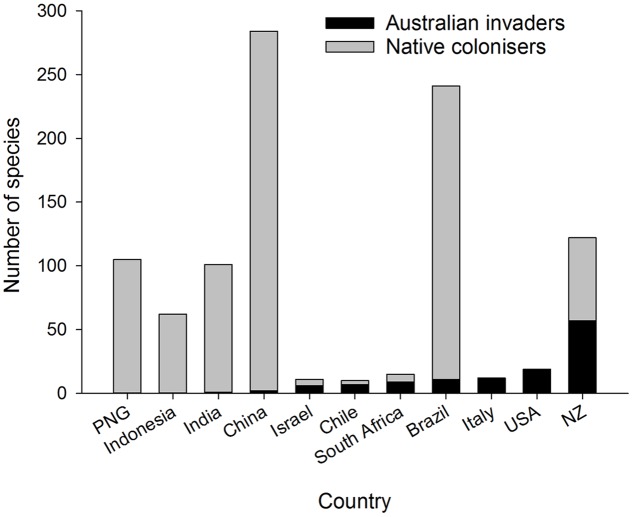
**Number of Australian invaders and native colonizers associated with eucalypts in different tropical and temperate countries**. The number of Australian invaders increases from left to right.

A well-recognized pattern in eucalypt communities outside Australia is the dominance of local species colonizing eucalypts in tropical zones, whereas in temperate zones introduced species of Australian origin dominate the herbivore community (Ohmart and Edwards, 1991) (**Figure 3**). For example, many species of native termites are associated with introduced eucalypts in South America, southern China and India (Constantino, 2002; Zhenghong, 2003; Nair, 2007). There are some insect genera and species common to Southeast Asia, Papua New Guinea and tropical Australia (Common, 1980; Wang, 1995) but very few endemic Australian insects have colonized eucalypts across Asia (Zhenghong, 2003) including China (Zhu et al., 2012; Yen et al., 2013) and India (Nair, 2007; Ramanagouda et al., 2010). The presence of either native eucalypts such as *E. deglupta* in Indonesia, East Timor, the Philippines and Papua New Guinea (Pryor et al., 1995) or other native Myrtaceae in South America, Africa and New Zealand may have pre-adapted local herbivores in those regions to colonize exotic eucalypts when the opportunity arose (Wingfield et al., 2008; Paine et al., 2011). Insect herbivores associated with tropical Asian eucalypts are potential native colonizers of Australian eucalypt species introduced into Asia and conversely are considered a biosecurity risk to Australian eucalypt forests (Wylie and Floyd, 2002). Brazil is another good example: it has a high diversity of native (non-eucalypt) Myrtaceae and associated native insect herbivores [>200 spp. of mostly leaf chewing Lepidoptera and Coleoptera (Zanuncio et al., 1994; dos Anjos and Majer, 2003)] plus leaf cutting ants, *Atta* spp. (Ferreira-Filho et al., 2015). Such native insects have transferred readily onto introduced eucalypts and now dominate the insect fauna associated with Brazilian eucalypt plantations (Zanuncio et al., 1994; Paine et al., 2011). New Zealand, however, has similar numbers of native colonizers and Australian invaders on eucalypts (Withers, 2001) (**Figure 3**) despite the presence of some Myrtaceae in the native flora. New Zealand’s insect herbivores may be less predisposed to colonize eucalypts because the New Zealand Myrtaceae are more distantly related to eucalypts than are the South American Myrtaceae (Ridley et al., 2000). Certainly geographic proximity, prevailing winds, and high trade volumes across the Tasman increase the likelihood of Australian insects reaching New Zealand (Fox, 1978; Sturman et al., 1997). North America and Europe have few native Myrtaceae and eucalypts in these regions are dominated by invasive Australian species (Paine et al., 2011).

**FIGURE 4:**
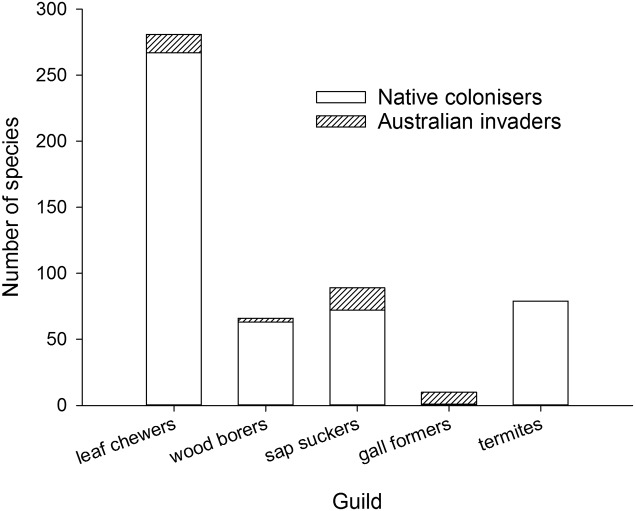
**Cumulative worldwide numbers of Australian invaders and native colonizers associated with eucalypts and grouped by guild**.

A closer look at the variety of herbivores found on eucalypts outside Australia suggests herbivore guilds may play a role in the construction of new eucalypt communities, something first considered in New Zealand by Withers (2001). Australian invaders can be classified into guilds easily but the relative proportion of native colonizers will be an underestimate for most guilds because there is not sufficient information to assign every native colonizer to a guild. For those native colonizers that could be identified (**Figure 4**), native leaf chewers, wood borers, termites and sap suckers are more dominant on eucalypts than Australian members of those guilds whereas gall formers are almost exclusively invasive Australian species (a gall former is associated with *E. deglupta* in the Philippines where both herbivore and eucalypt are native, Speight and Wylie, 2001). Gall formers form highly specific relationships with their host plants (Raman et al., 2005) relative to the other guilds considered here and this host specificity may act to constrain colonization of eucalypts by native gall forming species. The lack of native gall forming colonizers on eucalypts may partly explain the extremely rapid invasion by the Australian gall former *L. invasa* because it took over a vacant niche in eucalypt communities worldwide. The sap sucking guild has a high proportion of native colonizers but also includes a suite of Australian invaders dominated by the eucalypt psyllids. The dominance of native colonizers in the leaf chewing and wood boring guilds, by pre-empting plant resources, may have made eucalypt plantations in Asia and South America less vulnerable to new Australian invaders from these guilds.

## Natural Enemies

**FIGURE 5:**
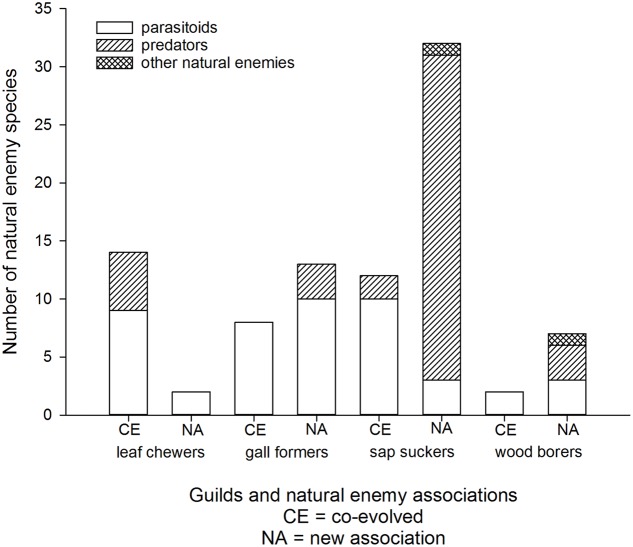
**Cumulative worldwide numbers of co-evolved and new association natural enemies of Australian eucalypt insects grouped by guild**.

Most eucalypt herbivores are associated with an extensive parasitoid fauna within their native ranges (e.g., Austin and Allen, 1989; Austin et al., 1994; Mendel et al., 2007). Escape from natural enemies is one factor expected to contribute to invasion success elsewhere (Liu and Stiling, 2006; Branco et al., 2014). Certainly invasive eucalypt insects are often targets for classical biological control (e.g., Faulds, 1991; Hodkinson, 1999; Tribe, 2000). Deliberate introductions of natural enemies (mostly host-specific parasitoids) are documented for eight of the eleven most invasive eucalypt herbivores and for at least another five species that are not as widespread (Garnas et al., 2012; Avila et al., 2013). Parasitoids are the most common co-evolved natural enemies associated with leaf chewers, gall formers, sap suckers, and wood borers, although there are some co-evolved predators associated with leaf chewers and sap suckers (**Figure 5**). It is important to recognize that not all Australian eucalypt herbivores cause significant damage once established in a new country and some remain at very low densities (Withers, 2001; Martinez et al., 2014). Planned biological control efforts have targeted those species that developed damaging populations.

**FIGURE 6:**
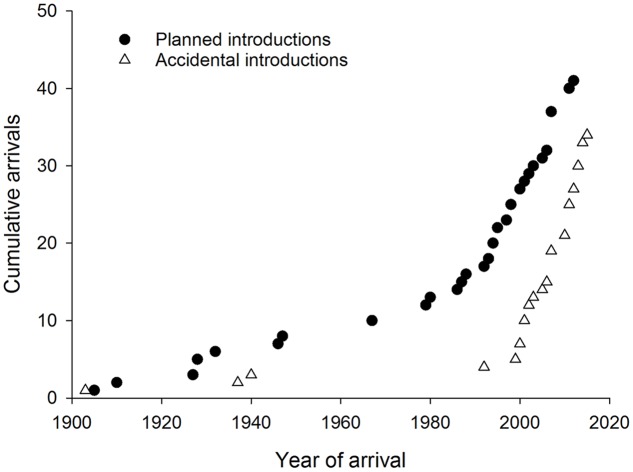
**Cumulative worldwide establishment of natural enemies associated with Australian eucalypt insects through planned (classical biological control) and accidental introductions**.

Accidental introductions of co-evolved natural enemies into new countries have increased sharply since 2000 following a similar trend to that in planned introductions worldwide (**Figure 6**). Natural dispersal after planned biological control programs resulted in some accidental introductions to adjacent countries (Costanzi et al., 2003; Doganlar and Mendel, 2007). In other cases the natural enemy has arrived accidentally either together with, or very soon after, the pest (Branco et al., 2009; Burks et al., 2015a). Two accidental introductions of Australian hyperparasitoids associated with eucalypt insects and their parasitoids have been reported from New Zealand (Jones and Withers, 2003; Berry, 2007) but introductions at the fourth trophic level have not been reported elsewhere yet. No reports of natural enemies (co-evolved or new associations) were found from the invasive geographic range of 20 Australian eucalypt herbivores (eight leaf chewers, six gall formers, five sap suckers and one wood borer). Most of these species have invaded very few countries with just one gall former, *Q. nova*, that is widespread outside Australia.

New associations may form between invasive eucalypt herbivores and local natural enemies in the affected region(s). Eucalypt sap suckers seem to have been colonized by a higher proportion of local natural enemies compared with the other herbivore guilds (**Figure 5**). This includes the two invasive sap suckers, *B. occidentalis* and *C. spatulata*, which do not have any co-evolved natural enemies reported from their invasive geographic range. The only records of parasitism on the sap sucker *B. occidentalis* outside of Australia are by an unidentified local parasitoid in China (Yen et al., 2013) and by the newly described parasitoid species *Psyllaephagus blastopsyllae* in Cameroon (Tamesse et al., 2014). The majority of these new associations with sap suckers are due to generalist predators adapting to a new food source. For example, local generalist predator species (hoverflies, lacewings, predatory bugs, etc) have been recorded feeding on *C. spatulata* in Brazil and Portugal (Valente et al., 2004; Santana and Burckhardt, 2007), on *Glycaspis brimblecombei* in California (Erbilgin et al., 2004) and on *T. peregrinus* in Argentina (Santadino et al., 2013). There is one reported example where an Australian pest has arrived in a new country (*T. peregrinus* in Portugal) in association with a new predator, the South American lacewing *Hemerobius bolivari* (Garcia et al., 2013). In Brazil the sap sucker *T. peregrinus* is also infected by an entomopathogenic fungus (Mascarin et al., 2012). The predator *Anthocoris nemoralis* attacks *G. brimblecombei* not only in the Palearctic region where the predator is native, but also in North America where this predator was introduced in the 1960s (Horton et al., 2004; Karaca et al., 2015).

New natural enemy associations recorded from the other herbivore guilds are linked with a single species in each case. The highly invasive gall former, *L. invasa*, is attacked by local parasitoid species in eight countries so far (Protasov et al., 2008; Doganlar et al., 2013; Udagedara and Karunaratne, 2014; Yang et al., 2014) and by several spider species in China (Zheng et al., 2014). Local parasitoids also attack the wood borer, *P. semipunctata*, in Morocco, California, and South Africa (Fraval and Haddan, 1988; Hanks et al., 1997; Prinsloo, 2004) while its eggs are subject to predation by ants in Portugal (Way et al., 1992). In Argentina *P. semipunctata* is also attacked by a nematode parasite (Achinelly and Camino, 2011). Two parasitoid species (one exotic, one presumed native) have formed new associations with the leaf chewer, *U. lugens*, in New Zealand (Mansfield et al., 2005). No new associations have been reported from the *Gonipterus* species complex, which are the most widespread leaf chewers.

When native insect herbivores colonize eucalypts, their natural enemies may follow them into this new habitat. This is best documented for the leaf chewing guild with very sparse records relating to natural enemies of native eucalypt colonizers in the wood borer (Noyes, 1990) and sap sucker (Speight and Wylie, 2001) guilds and no records at all for gall formers or termites. At least 12 native lepidopteran species (leaf chewers) feeding on introduced eucalypts have been associated with one or more natural enemies, mostly parasitoids. The majority of records are from Brazilian eucalypt plantations (e.g., de Campos and Cure, 1992; de Oliveira et al., 2000; Macedo-Reis et al., 2013) but there are also examples from East Africa, India, and Malaysia (Okelo, 1972; Joshi et al., 1988; Speight and Wylie, 2001). The predatory pentatomids (*Podisus* spp.) are the only co-evolved predators that attack native caterpillars colonizing eucalypts in Brazil, and are themselves attacked by several local species of egg parasitoid that have followed their hosts into the new eucalypt habitat (Zanuncio et al., 2000).

New associations have been recorded between two native Brazilian leaf chewers found on eucalypts, *Thyrinteina arnobia* and *Melanolophia consimilaria*, and the exotic parasitoid, *Trichospilus diatraeae*. This eulophid parasitoid was introduced to the Caribbean originally as a biological control agent for a sugarcane pest but it also attacks lepidopteran pests of other crops (Bennett et al., 1987). Its arrival in Brazil is presumed to be accidental (Pereira et al., 2008; Zache et al., 2010).

## Conclusion

The earliest Australian invaders began to spread globally in the late 1800s as eucalypt forestry developed outside of Australia. Australian eucalypt insects have continued to spread ever since. Some guilds (sap suckers, gall formers) have contributed more highly invasive species than others (wood borers, leaf chewers). Native colonizers, particularly leaf chewers, have also moved onto introduced eucalypts, increasing the diversity of new eucalypt communities. This trend is strongest in the tropical regions where biodiversity is high and the pool of potential native colonizers is largest. The observed patterns of invasion among different guilds suggest a new hypothesis: eucalypt communities that accumulate higher numbers of native colonizers from a particular guild (e.g., leaf chewers) will have greater biotic resistance to new invasions by Australian species from that same guild. It is challenging to test this hypothesis explicitly because it requires evidence not only of successful and failed invasions but also some measure of inter-specific competition within new eucalypt communities. Successful invasions are usually documented, as this review demonstrates, but data on border interceptions of particular insect groups are less readily available. The effects of competition between native colonizers and Australian invaders on invasion success warrants further investigation.

Biological control by co-evolved natural enemies can reduce the damage caused by Australian invaders, as the history of successful classical biological control programs shows. The impact of co-evolved natural enemies does not appear to prevent the spread of Australian invaders into new regions. Native natural enemies that form new associations with Australian invaders may also contribute to biological control, particularly for Australian sap suckers, which have a higher proportion of new associations than other guilds. Co-evolved natural enemies may contribute to control of native colonizers on eucalypts although the literature is still sparse on this point. Eucalypt communities outside Australia can now support unplanned invasions of the third, and occasionally even the fourth trophic levels. The increasing numbers of Australian invaders that have arrived simultaneously with their natural enemy demonstrate this point. The complexity of these new eucalypt communities is expected to increase in future.

## Author Contributions

The author confirms being the sole contributor of this work and approved it for publication.

## Conflict of Interest Statement

The author declares that the research was conducted in the absence of any commercial or financial relationships that could be construed as a potential conflict of interest.

## Supplementary Material

DATA SHEET 1 **Database of Australian invaders, native colonisers, and their associated natural enemies**.
