# Supplementary material for: New Communities on Eucalypts Grown Outside Australia
Source: Front Plant Sci. 2016 Nov 29;7:1812. doi: 10.3389/fpls.2016.01812 (PMC5126142; doi:10.3389/fpls.2016.01812)
Supplement: Supplementary file 2 [file Data_Sheet_2.DOCX]

***Supplementary Material***

**New communities on eucalypts grown outside Australia**

**Sarah Mansfield**

sarah.mansfield@agresearch.co.nz

**Bibliography of supporting references not cited in main text.**

Alves JB, Zanuncio JC, Forlin A, Piffer AA. (1994) Faunistic analysis and population fluctuations of Lepidoptera associated with *Eucalyptus* in Niquelandia, Goias State, Brazil. Revista Arvore 18: 159-168.

Aquino DA, Botto EN, Loiacono MS, Pathauer P. (2011) The wasp of the eucalyptus gall, *Leptocybe invasa* Fischer & La Salle (Hymenoptera: Eulophidae: Tetrastichinae), in Argentina. Revista de Investigaciones Agropecuarias 37: 159-164.

Avila GA, Berndt LA. (2011) Release of a new biological control agent, *Cotesia urabae*, against *Uraba lugens* in New Zealand. New Zealand Plant Protection 64: 282-294.

Aytar F, Dagdas S, Duran C. (2011) Australian Insects Affecting Eucalyptus Species in Turkey. Silva Lusitana 19: 41-47.

Bain J, Kay MK. (1989) *Paropsis charybdis* Stal, eucalyptus tortoise beetle (Coleoptera: Chrysomelidae). In: Cameron PJ, Hill RL, Bain J, Thomas WP (Eds*.*) A review of biological control of invertebrate pests and weeds in New Zealand 1874 to 1987. Oxon, CABI & DSIR. pp. 281-287.

Barreto MR, Mojena PA. (2014) Registration of *Thyrinteina arnobia arnobia* (Stoll) (Lepidoptera: Geometridae) in *Eucalyptus* sp. (Myrtaceae) in Sorriso, Mato Grosso and its predation by *Zelus armillatus* (Lepeletier & Serville) (Hemiptera: Reduviidae: Harpactorinae). EntomoBrasilis 7: 69-71.

Bouvet JPR, Harrand L, Burckhardt D. (2005) First record of *Blastopsylla occidentalis* and *Glycaspis brimblecombei* (Hemiptera: Psyllidae) from Argentina. Revista de la Sociedad Entomologica Argentina 64: 99-102.

Branco M, Franco JC, Valente C, Mendel Z. (2006) Survey of *Eucalyptus* gall wasps (Hymenoptera: Eulophidae) in Portugal. Boletin de Sanidad Vegetal Plagas 32: 199-202.

Bueno VHP, Berti Filho E, Matioli JC. (1987) Aspects of the biology and behaviour of *Nesolynx* sp. (Hymenoptera, Eulophidae). Anais da Escola Superior de Agricultura Luiz de Queiroz 44: 105-117.

Burckhardt D, Elgueta M. (2000) *Blastopsylla occidentalis* Taylor (Hemiptera: Psyllidae), a new introduced eucalypt pest in Chile. Revista Chilena de Entomologia 26: 57-61.

Caleca V, Lo Verde G, Rizzo MC, Rizzo R. (2011) Dispersal rate and parasitism by *Closterocerus chamaeleon* (Girault) after its release in Sicily to control *Ophelimus maskelli* (Ashmead) (Hymenoptera, Eulophidae). Biological Control 57: 66-73.

Chirinzane CJ, de Souza MD, Sousa NJ, Rezende EH, Alves T, Bandeira RR, Fernandes A dos M. (2014) First report of *Leptocybe invasa* Fisher and La Salle (Hymenoptera: Eulophidae) in Mozambique. African Journal of Agricultural Research 9: 3555-3558.

Cocquempot C, Malausa J-C, Thaon M, Brancaccio L. (2012) The Red Gum Lerp Psyllid (*Glycaspis brimblecombei* Moore) introduced on French eucalyptus trees (Hemiptera, Psyllidae). Bulletin de la Societe Entomologique de France 117: 363-370.

Cowles RS, Downer JA. (1995) Eucalyptus snout beetle detected in California. California Agriculture 49: 38-40.

Crouzel ISD, Saini ED, Sonvico V, Botto EN. (1985) *Pyrrhopyge pelota* Plotz (Lep. Hesperiidae). Morphological study of the immature stages. Revista de Investigaciones Agropecuarias 16: 171-195.

Daane KM, Sime KR, Dahlsten DL, Andrews JW, Zuparko RL. (2005) The biology of *Psyllaephagus bliteus* Riek (Hymenoptera: Encyrtidae), a parasitoid of the red gum lerp psyllid (Hemiptera: Psylloidea). Biological Control 32: 228-235.

Dahlsten DL, Rowney DL, Copper WA. (2000) Landscapes: integrating biological control of the eugenia psyllid into the day-to-day management of the landscape in Disneyland. In: Hoddle MS *ed.* California Conference on Biological Control II. The Historic Mission Inn, Riverside, California, USA. pp. 59-64

Dahlsten DL, Rowney DL, Copper WA, Tassan RL, Chaney WE, Robb KL, Tjosvold S, Bianchi M, Lane P. (1998) Parasitoid wasp controls blue gum psyllid. California Agriculture 52: 31-34.

Dahlsten DL, Rowney DL, Robb KL, Downer JA, Shaw DA, Kabashima JN. (2003) Biological control of introduced psyllids on eucalyptus. In: Van Driesche RG *ed.* Proceedings of the 1st International Symposium on Biological Control of Arthropods. Honolulu, Hawaii, USA, pp. 356-361.

De Marzo L. (2007) The parasitoid *Closterocerus chamaeleon* (Girault) reported from Basilicata and Apulia (Hymenoptera Eulophidae). Bollettino di Zoologia Agraria e di Bachicoltura 39: 231-237.

Degefu DT, Hurley BP, Garnas J, Wingfield MJ, Ahumada R, Slippers B. (2013) Parallel host range expansion in two unrelated cossid moths infesting *Eucalyptus nitens* on two continents. Ecological Entomology 38: 112-116.

Dhahri S, Ben Jamaa ML, Garcia A, Boavida C, Branco M. (2014) Presence of *Glycaspis brimblecombei* and its parasitoid *Psyllaephagus bliteus* in Tunisia and Portugal. Silva Lusitana 22: 99-115.

Di-Iorio OR. (2004) Exotic species of Cerambycidae (Coleoptera) introduced in Argentina. Part 1. The genus *Phoracantha* Newman, 1840. Agrociencia 38: 503-515.

Dittrich-Schroeder G, Harney M, Neser S, Joffe T, Bush S, Hurley BP, Wingfield MJ, Slippers B. (2014) Biology and host preference of *Selitrichodes neseri*: A potential biological control agent of the Eucalyptus gall wasp, *Leptocybe invasa*. Biological Control 78: 33-41.

Dourojeanni MJ. (1967) *Phoracantha semipunctata*, newly recorded in Peru. Revista forestal Peruviana 1: 3-11.

El Nasr AS, Abd-Rabou S. (2012) Common pests of psyllids and whiteflies (Hemiptera: Psylloidea: Aleyrodoidea) infesting orchard trees in Egypt. Egyptian Academic Journal of Biological Sciences 5: 147-152.

Ferreira Filho PJ, Wilcken CF, de Oliveira NC, Dal Pogetto MHFD, Vianna Lima AC. (2008) Population dynamics of red gum lerp psyllid, *Glycaspis brimblecombei* (Moore, 1964) (Hemiptera: Psyllidae) and its parasitoid, *Psyllaephagus bliteus* (Hymenoptera: Encyrtidae), in *Eucalyptus camaldulensis* plantation. Ciencia Rural 38: 2109-2114.

Fidalgo P, Olivares TS, de Haro AM, Baranao JJ. (2005) Biological control of *Ctenarytaina eucalypti* (Hem.: Psyllidae), pest of eucalyptus in Argentina. Bosque 26: 91-93.

Garcıa RR, Somoano A, Moreno A, Burckhardt D, de Queiroz DL, Minarro M. (2014) The occurrence and abundance of two alien eucalypt psyllids in apple orchards. Pest Management Science 70: 1676-1683.

Gess FW. (1964) The discovery of a parasite of the *Phoracantha* beetle (Coleoptera: Cerambycidae) in the Western Cape. Journal of the Entomological Society of Southern Africa 27: 152.

Guedes RNC, Zanuncio TV, Zanuncio JC, Medeiros AGB. (2000) Species richness and fluctuation of defoliator Lepidoptera populations in Brazilian plantations of *Eucalyptus grandis* as affected by plant age and weather factors. Forest Ecology and Management 137: 179-184.

Halperin J. (1961) Pests and diseases of Eucalypts in Israel. In: Contributions on Eucalypts in Israel. Ilanot, National & University Institute of Agriculture. pp. 19-22.

Halperin J. (1963) Injury to Eucalypts caused by *Achradidius creticus* Kies. and *Opatroides punctulatus* Bruelle. In: Contributions on Eucalypts in Israel. Ilanot, National & University Institute of Agriculture. pp. 43-47.

Hanks LM, Millar JG, Paine TD, Campbell CD. (2000) Classical biological control of the Australian weevil *Gonipterus scutellatus* (Coleoptera: Curculionidae) in California. Environmental Entomology 29: 369-375.

Hanks LM, Paine TD, Millar JG. (1996) Tiny wasp helps protect eucalypts from eucalyptus longhorned borer. California Agriculture 50: 14-16.

Harish K, Ramanagouda SH, Vastrad AS, Basavanagoud K. (2010) Status of the eucalyptus gall wasp, *Leptocybe invasa* Fisher & La Salle, and its native parasitoids in Karnataka. Pest Management and Economic Zoology 18: 78-84.

Hepburn GA, Loedolff J. (1964) A new pest in South Africa (*Neocleora herbuloti* Fletcher, order Lepidoptera, family Geometridae). Forestry in South Africa 4: 1-18.

Hernandez Carmen M, Aquino DA, Cuello EM, Andorno AV, Botto EN. (2015) First record of *Megastigmus zebrinus* Grissell from Argentina (Hymenoptera: Torymidae) associated with galls of *Leptocybe invasa* (Hymenoptera: Eulophidae). Revista de la Sociedad Entomologica Argentina 74: 75-77.

Hodkinson ID. (1991) The Australian psyllid *Blastopsylla occidentalis* Taylor Homoptera Psylloidea on *Eucalyptus* Myrtaceae in Mexico. Pan-Pacific Entomologist 67: 72.

Huerta A, Jaramillo J, Araya JE. (2011) Establishment of the red gum psyllid parasitoid *Psyllaephagus bliteus* on Eucalyptus in Santiago, Chile. Forest Systems 20: 339-347.

Ide SM, Ruiz CG, Sandoval AC, Valenzuela JE. (2011) Detection of *Thaumastocoris peregrinus* (Hemiptera: Thaumastocoridae) associated to *Eucalyptus* spp. in Chile. Bosque 32: 309-313.

Jagdish C, Chander J. (2011) Studies on the management of Eucalyptus gall wasp *Leptocybe invasa* Fisher and La Salle in Haryana. Indian Journal of Ecology 38: 235.

Jones ME, Daane KM, Paine TD. (2011) Establishment of *Psyllaephagus parvus* and *P. perplexans* as serendipitous biological control agents of Eucalyptus psyllids in southern California. Biocontrol 56: 735-744.

Joyce AL, Hanks LM, Paine TD, Millar JG. (2000) Effect of host larval size on sex ratio of progeny of *Syngaster lepidus* (Hymenoptera: Braconidae) attacking *Phoracantha semipunctata* (Coleoptera: Cerambycidae) and *P. recurva* borers on *Eucalyptus camaldulensis*. In: Hoddle MS *ed.* California Conference on Biological Control II. The Historic Mission Inn, Riverside, California, USA. pp. 151-154.

Kawabe Y, Ito K. (2003) Disease and insect pest damage in afforested areas on acid sulfate soil in the Mekong Delta, Vietnam. Tropical Forestry 57: 25-33.

Kim I-K, McDonald M, La Salle J. (2005) *Moona*, a new genus of tetrastichine gall inducers (Hymenoptera: Eulophidae) on seeds of *Corymbia* (Myrtaceae) in Australia. Zootaxa 989: 1-10.

Kim I-K, Mendel Z, Protasov A, Blumberg D, La Salle J. (2008) Taxonomy, biology, and efficacy of two Australian parasitoids of the eucalyptus gall wasp, *Leptocybe invasa* Fisher & La Salle (Hymenoptera: Eulophidae: Tetrastichinae). Zootaxa 1910: 1-20.

Kurylo CL, Garcia MS, Costa VA, Tibola C, Ramiro GA, Finkenauer E. (2010) Occurrence of *Ctenarytaina eucalypti* (Maskell) (Hemiptera: Psyllidae) and its Natural Enemy *Psyllaephagus pilosus* Noyes (Hymenoptera: Encyrtidae) in *Eucalyptus globulus* in the State of Rio Grande do Sul, Brazil. Neotropical Entomology 39: 671-673.

La Salle J, Arakelian G, Garrison RW, Gates MW. (2009) A new species of invasive gall wasp (Hymenoptera: Eulophidae: Tetrastichinae) on blue gum (*Eucalyptus globulus*) in California. Zootaxa 2121: 35-43.

Lanfranco D, Dungey HS. (2001) Insect damage in *Eucalyptus*: A review of plantations in Chile. Austral Ecology 26: 477-481.

Laudonia S. (2006) A new psyllid on eucalyptus. Informatore Agrario 62: 89.

Laudonia S, Sasso R. (2012) The bronze bug *Thaumastocoris peregrinus*: a new insect recorded in Italy, damaging to *Eucalyptus* trees. Bulletin of Insectology 65: 89-93.

Longo S. (2009) The impact of introduced exotic insect plant pests to the Italian forest ecosystems. Atti della Accademia Nazionale Italiana di Entomologia Rendiconti 57: 69-77.

Maatouf N, Lumaret J-P. (2012) Eco-ethology of new invasive pest species on eucalyptus plantation of Morocco. Annales De La Societe Entomologique De France 48: 289-297.

Mafia RG, da Silva JB, Ramos JF. (2013) Characterization of damages caused by *Heilipodus naevulus* in eucalypt plantations on Espirito Santo, Brazil. Ciencia Rural 43: 258-261.

Mafia RG, Mendes P, Corassa JE, De Nadai J. (2014) Comparative analysis of outbreaks and damages caused by leaf beetles *Costalimaita ferruginea* (Fabricius, 1801) and *Costalimaita lurida* (Lefevre, 1891) (Coleoptera: Chrysomelidae) in eucalypt plantations. Revista Arvore 38: 829-836.

Malausa JC, Girardet N. (1997) Biological control of the blue gum psyllid. Acclimatization on the Cote d'Azur of a promising beneficial, *Psyllaephagus pilosus*. Phytoma 50: 49-51.

Martinez G, Bianchi M. (2010) First record in Uruguay of the bronze bug, *Thaumastocoris peregrinus* Carpintero and Dellappé, 2006 (Heteroptera: Thaumastocoridae). Agrociencia 14: 15-18.

Martinez G, Gomez D, Taylor GS. (2014) First record of the Australian psyllid *Blastopsylla occidentalis* Taylor (Hemiptera, Psylloidea) from Uruguay. Transactions of the Royal Society of South Australia 138: 231-236.

Mifsud D. (2012) *Leptocybe invasa* Fisher & La Salle, 2004 and *Ophelimus maskelli* Haliday, 1844-two new records of gall forming Eulophidae from Malta (Hymenoptera, Chalcidoidea). Bulletin of the Entomological Society of Malta 5: 189-193.

Miller D, Clark AF. (1935) Control of forest insect pests. Distribution of parasites in New Zealand. New Zealand Journal of Science and Technology 16: 301-307.

Milonas PG, Partsinevelos GK. (2014) First record of *Glycaspis brimblecombei* Moore, 1964 (Hemiptera: Psyllidae) in Greece. Hellenic Plant Protection Journal 7: 19-23.

Montemayor SI, Dellape PM, Melo MC. (2015) Geographical distribution modelling of the bronze bug: a worldwide invasion. Agricultural and Forest Entomology 17: 129-137.

Morales CF, Bain J. (1989) *Eriococcus coriaceus* Maskell, gum tree scale (Homoptera: Eriococcidae). In: P. J. Cameron RLH, J. Bain and W. P. Thomas *eds.* A review of biological control of invertebrate pests and weeds in New Zealand 1874-1987. Oxon, UK, CAB International and DSIR. pp. 263-266.

Mukhtar A, Ahmed M. (1989) Feeding diversity of *Myllocerus viridanus* Fab. (Coleoptera: Curculionidae) from south India. Indian Forester 115: 832-838.

Nadel RL, Noack AE. (2012) Current understanding of the biology of *Thaumastocoris peregrinus* in the quest for a management strategy. International Journal of Pest Management 58: 257-266.

Nasu Y, Arita Y, Kimura M, Ogata A. (2004) Some lepidopterous pests of eucalyptus trees from Japan. Japanese Journal of Applied Entomology and Zoology 48: 123-133.

Naude TJ. (1952) Annual report for the Department of Agriculture South Africa for the year ended 31 August 1952. Entomological services and research. Farming in South Africa 27: 616-621.

Naumann ID. (1991) Revision of the Australian genus *Enoggera* Girault (Hymenoptera: Pteromalidae: Asaphinae). Journal of the Australian Entomological Society 30: 1-17.

Nuttall MJ. (1989) *Gonipterus scutellatus* Gyllenhal gum tree weevil (Coleoptera: Curculionidae). In: Cameron PJ, Hill RL, Bain J, Thomas WP *eds.* A review of biological control of invertebrate pests and weeds in New Zealand 1874-1987. Oxon, UK, CAB International and DSIR. pp. 267-270.

Nyeko P, Mutitu EK, Day RK. (2009) *Eucalyptus* infestation by *Leptocybe invasa* in Uganda. African Journal of Ecology 47: 299-307.

Nyeko P, Mutitu KE, Otieno BO, Ngae GN, Day RK. (2010) Variations in *Leptocybe invasa* (Hymenoptera: Eulophidae) population intensity and infestation on eucalyptus germplasms in Uganda and Kenya. International Journal of Pest Management 56: 137-144.

Ohashi OS, Berti Filho E. (1988) Natural enemies of *Eupseudosoma aberrans* and *E. involuta* (Lepidoptera, Arctiidae), pests of *Eucalyptus* spp. (Myrtaceae). Instituto de Pesquisas e Estudos Florestais 40: 43-44.

Oliveira HN, Zanuncio TV, Zanuncio JC, Serrao JE. (2008) The eucalypt defoliator *Thyrinteina arnobia* (Lepidoptera: Geometridae) protects its eggs from parasitism. Biological Letters 45: 23-28.

Pastori PL, Pereira FF, Andrade GS, Silva RO, Zanuncio JC, Pereira AIA. (2012) Reproduction of *Trichospilus diatraeae* (Hymenoptera: Eulophidae) in pupae of two lepidopterans defoliators of eucalypt. Revista Colombiana De Entomologia 38: 91-93.

Pereira FF, Zanuncio TV, Zanuncio JC, Pratissoli D, Tavares MT. (2008) Species of Lepidoptera defoliators of *Eucalyptus* as new host for the parasitoid *Palmistichus elaeisis* (Hymenoptera: Eulophidae). Brazilian Archives of Biology and Technology 51: 259-262.

Pereira JMM, Zanuncio JC, Schoereder JH. (1994) Faunistics indexes of the major lepidopterous pests in the Lassance and Sao Bento Abade Regions, in Minas Gerais, Brazil. Revista Arvore 18: 79-86.

Pereira JMM, Zanuncio JC, Schoereder JH, Candido do Nascimento E. (1995) Faunistic indices of major Lepidoptera damaging Eucalyptus in the Cacpava and Sao Jose dos Campos Regions, Sao Paulo. Revista Brasileira de Entomologia 39: 447-452.

Pereira JMM, Zanuncio JC, Schoereder JH, Gasperazzo WL. (1994) Faunistic indexes and population dynamics of major Lepidopterous pests of Eucalyptus in Montes Claros Region, Minas Gerais State. Anais da Sociedade Entomologica do Brasil 23: 327-334.

Pereira JMM, Zanuncio TV, Zanuncio JC, Pallini A. (2001) Lepidoptera pests collected in *Eucalyptus urophylla* (Myrtaceae) plantations during five years in Tres Marias, State of Minas Gerais, Brazil. Revista De Biologia Tropical 49: 1073-1082.

Perez-Otero R, Mansilla JP, Borrajo P, Ruiz F. (2011) First record of *Blastopsylla occidentalis* Taylor (Homoptera: Psyllidae) in the Iberian Peninsula. Boletin de Sanidad Vegetal Plagas 37: 139-144.

Petro R, Madoffe SS, Iddi S. (2014) Infestation density of eucalyptus gall wasp, *Leptocybe invasa* Fisher and La Salle (Hymenoptera: Eulophidae) on five commercially grown *Eucalyptus* species in Tanzania. Journal of Sustainable Forestry 33: 276-297.

Pham Quang T, Dell B, Burgess TI. (2009) Susceptibility of 18 eucalypt species to the gall wasp *Leptocybe invasa* in the nursery and young plantations in Vietnam. Scienceasia 35: 113-117.

Puker A, Moron MA, De Oliveira Junior O, Message D. (2011) First record of *Leucothyreus albopilosus* (Coleoptera: Scarabaeidae: Rutelinae: Geniatini) in lesions on stem of *Eucalyptus citriodora* and beehives of *Apis mellifera*. Entomological Science 14: 230-233.

Purvis G, Dunne R, Chauzat MP. (1998) Biological control of eucalyptus psyllid. Farm & Food 8: 24-25.

Reguia K, Peris-Felipo FJ. (2013) *Glycaspis brimblecombei* Moore, 1964 (Hemiptera Psyllidae) invasion and new records in the Mediterranean area. Biodiversity Journal 4: 501-506.

Ribeiro Dias TK, Wilcken CF, Soliman EP, Gil-Santana HR, Zaché B. (2012) Occurrence of *Atopozelus opsimus* preying on nymphs and adults of *Glycaspis brimblecombei*. Phytoparasitica 40: 137-141.

Rivera AC, Carbone SS, Andres JA. (1999) Life cycle and biological control of the *Eucalyptus* snout beetle (Coleoptera, Curculionidae) by *Anaphes nitens* (Hymenoptera, Mymaridae) in north-west Spain. Agricultural and Forest Entomology 1: 103-109.

Rodas CA, Serna R, Hurley BP, Bolanos MD, Granados GM, Wingfield MJ. (2014) Three new and important insect pests recorded for the first time in Colombian plantations. Southern Forests 76: 245-252.

Rodriguez FA, Saiz FG. (2006) Parasitoidism of *Psyllaephagus pilosus* Noyes (Hym.: Encyrtidae) on the blue gum psyllid, *Ctenarytaina eucalypti* (Maskell) (Hem.: Psyllidae) in V region eucalypts plantations. Agricultura Tecnica 66: 342-351.

Sangtongpraow B, Charernsoml K, Siripatanadilok S. (2011) Longevity, Fecundity and Development Time of Eucalyptus Gall Wasp, *Leptocybe invasa* Fisher & La Salle (Hymenoptera: Eulophidae) in Kanchanaburi Province, Thailand. Thai Journal of Agricultural Science 44: 155-163.

Santos A, Zanetti R, Almado RP, Zanuncio JC. (2014) Cerambycidae associated with hybrid *Eucalyptus urograndis* and native vegetation in Carbonita, Minas Gerais State, Brazil. Florida Entomologist 97: 523-527.

Schedl W. (1999) Invasion of the Eucalyptus borer, *Phoracantha semipunctata* (F.), in the Mediterranean Basin and at the Canary Islands (Coleoptera: Cerambycidae). Journal of Pest Science 72: 37-40.

Schnee H, Voigt D, Kaufer B. (2006) Biological control of the blue gum psyllid *Ctenarytaina eucalypti* (Maskell) (Hemiptera, Psyllidae) by the encyrtid *Psyllaephagus pilosus* Noyes (Hymenoptera, Encyrtidae) a success not only in California and Western Europe but also in Saxony. Gesunde Pflanzen 58: 99-106.

Souza GK, Pikart TG, Pikart FC, Serrão JE, Wilcken CF, Zanuncio JC. (2012) First record of a native heteropteran preying on the introduced eucalyptus pest, *Thaumastocoris peregrinus* (Hemiptera: Thaumastocoridae), in Brazil. Florida Entomologist 95: 517-520.

Spodek M, Burckhardt D, Protasov A, Mendel Z. (2015) First record of two invasive eucalypt psyllids (Hemiptera: Psylloidea) in Israel. Phytoparasitica 43: 401-406.

Tamesse JL, Laurentine S, Wenceslas Y, Joly DV. (2010) First record of *Blastopsylla occidentalis* Taylor, 1985 (Hemiptera: Psyllidae), a *Eucalyptus* psyllid in Cameroon, Central Africa. Entomological Research 40: 211-216.

Tang C, Wan X-J, W F-H, B S-X, Zheng-Qiang P. (2008) The blue gum chalcid, *Leptocybe invasa*, invaded Hainan province. Chinese Bulletin of Entomology 45: 967-971.

Taylor KL. (1997) A new Australian species of *Ctenarytaina* Ferris and Klyver (Hemiptera: Psyllidae: Spondyliaspidinae) established in three other countries. Australian Journal of Entomology 36: 113-115.

Tillyard RJ. (1931) The Control of the Eucalyptus Weevil (*Gonipterus*) by Parasites in South Africa and New Zealand. Journal of the Council for Scientific and Industrial Research, Australia 4: 57-58.

Tobi DR, Grehan JR, Parker BL. (1993) Review of the ecological and economic significance of forest Hepialidae (Insecta: Lepidoptera). Forest Ecology and Management 56: 1-12.

Torres JB, Zanuncio JC, Pratissoli D. (2001) Egg mortality factors of *Podisus nigrispinus* (Heteroptera: Pentatomidae) in *Eucalyptus cloeziana* plantations. Naturalia (Sao Paulo) 26: 245-256.

Tribe GD, Cillie JJ. (2000) Biological control of the *Eucalyptus*-defoliating Australian tortoise beetle *Trachymela tincticollis* (Blackburn) (Chrysomelidae: Chrysomelini: Paropsina) in South Africa by the egg parasitoid *Enoggera reticulata* Naumann (Hymenoptera: Pteromalidae: Asaphinae). African Entomology 8: 15-22.

Tune G-S, La Salle J. (2010) Pest Alert-a Newly Discovered Invasion of Gall-forming Wasps, *Leptocybe invasa* (Fisher & La Salle), on *Eucalyptus* Trees in Taiwan. Formosan Entomologist 30: 241-244.

Valdebenito BC, Sandoval SP, Esquivel EA, Acuña EA, Espinosa MA, Cancino JO, Rubilar RA, Muñoz FE, Cerda LA. (2009) First report of *Ectinogonia buquetti* (Spin.) (Coleoptera: Buprestidae) in bioenergy plantations of *Eucalyptus camaldulensis* (Dehnh.) in Chile. New Forests 38: 241-244.

Valente C, Hodkinson I. (2009) First record of the Red Gum Lerp Psyllid, *Glycaspis brimblecombei* Moore (Hem.: Psyllidae), in Europe. Journal of Applied Entomology 133: 315-317.

Vieira JM, Querino RB, Zucchi RA. (2014) On the identity of *Trichogramma demoraesi* Nagaraja (Hymenoptera: Trichogrammatidae), with a checklist and a key to *Trichogramma* species associated with *Erinnyis ello* (L.) (Lepidoptera, Sphingidae) in Brazil. Zootaxa 3869: 83-89.

Webb DVV. (1953) An ecological study of the wattle looper. Farming in South Africa 28: 385-390.

Wilcken CF, Soliman EP, Nogueira de Sá LA, Barbosa LR, Dias TKR, Ferreira-Filho PJ, Oliveira RJR. (2010) Bronze bug, *Thaumastocoris peregrinus* Carpintero and Dellape (Hemiptera: Thaumastocoridae) on *Eucalyptus* in Brazil and its distribution. Journal of Plant Protection Research 50: 201-205.

Williams JR, Moutia LA, Hermelin PR. (1951) The biological control of *Gonipterus scutellatus* Gyll. (Col. Curculionldae) in Mauritius. Bulletin of Entomological Research 42: 23-28.

Wistrom C, Sisterson MS, Pryor MP, Hashim-Buckey JM, Daane KM. (2010) Distribution of Glassy-Winged Sharpshooter and Threecornered Alfalfa Hopper on Plant Hosts in the San Joaquin Valley, California. Journal of Economic Entomology 103: 1051-1059.

Withers TM, Raman A, Berry JA. (2000) Host range and biology of *Ophelimus eucalypti* (Gahan) (Hym. Eulophidae), a pest of New Zealand eucalypts. New Zealand Plant Protection 53: 339-344.

X-H Yang, Yu Y-H, Wu Y-J, Qin J-L, Luo Y-Q. (2013) First report of *Endoclita signifer* (Lepidoptera: Hepialidae) as a new pest on *Eucalyptus*. Journal of Economic Entomology 106: 866-873.

Zache B, Wilcken CF, da Costa Zaché RR, de Souza NM. (2012) New occurrence of *Trichospilus diatraeae* Cherian & Margabandhu, 1942 (Hymenoptera: Eulophidae) as a parasitoid of *Spodoptera cosmioides* Walker, 1858 (Lepidoptera: Noctuidae) in Brazil. Biota Neotropica 12: 319-322.

Zache B, Zache RRC, Tavares MT, Wilcken CF. (2012) *Brachymeria pandora* (Crawford) (Hymenoptera: Chalcididae) as a New Parasitoid of *Thyrinteina leucocerae* (Rindge) (Lepidoptera: Geometridae) in Brazil. Neotropical Entomology 41: 343-344.

Zanuncio JC, do Nascimento EC, Camargo FRA, Zanuncio TV. (1994) Lepidoptera associated with *Eucalyptus* plantations in Cacapava and Sao Jose dos Campos, Sao Paulo state, Brazil. Cerne 1: 78-94.

Zanuncio JC, Fagundes M, Araujo MSS, Evaristo FDC. (1992) Monitoring Lepidoptera associated with eucalyptus plantations of the Acailandia region (state of Maranhao, Brazil) in the period August 1990 to July 1991. Acta Amazonica 22: 615-622.

Zanuncio JC, Jusselino-Filho P, Ribeiro RC, Castro AA, Zanuncio TV, Serrao JE. (2013) Fertility and Life Expectancy of a Predatory Stinkbug to Sublethal Doses of a Pyrethroid. Bulletin of Environmental Contamination and Toxicology 90: 39-45.

Zanuncio JC, Santos GP, dos Anjos N, Zanuncio TV. (1990) *Timocratica palpalis* Zeller 1839 Lepidoptera Stenomatidae Myrtaceae borer attacking *Eucalyptus saligna* in Minas Gerais Brazil. Anais da Sociedade Entomologica do Brasil 19: 465-470.

Zanuncio JC, Santos GP, Zanuncio TV, Smith MRB. (1994) Monitoring Lepidoptera associated with Eucalyptus: VII. Belo Oriente Region, Minas Gerais State, Brazil, from June, 1988, through May, 1989. Cientifica (Jaboticabal) 21: 361-371.
